# The central nervous system transcriptome of the weakly electric brown ghost knifefish (*Apteronotus leptorhynchus*): *de novo* assembly, annotation, and proteomics validation

**DOI:** 10.1186/s12864-015-1354-2

**Published:** 2015-03-11

**Authors:** Joseph P Salisbury, Ruxandra F Sîrbulescu, Benjamin M Moran, Jared R Auclair, Günther KH Zupanc, Jeffrey N Agar

**Affiliations:** Barnett Institute, Department of Chemistry and Chemical Biology, Northeastern University, 360 Huntington Avenue, 412 TF, Boston, MA 02115 USA; Laboratory of Neurobiology, Department of Biology, Northeastern University, 360 Huntington Avenue, 134 Mugar Life Sciences, Boston, MA 02115 USA; Department of Pharmaceutical Sciences, Northeastern University, 360 Huntington Avenue, 412 TF, Boston, MA 02115 USA

**Keywords:** De novo transcriptome, RNA-Seq, Brain, Spinal cord, *Apteronotus leptorhynchus*, Brown ghost knifefish, Proteomics, Non-coding RNA, Splice-junction peptides

## Abstract

**Background:**

The brown ghost knifefish (*Apteronotus leptorhynchus*) is a weakly electric teleost fish of particular interest as a versatile model system for a variety of research areas in neuroscience and biology. The comprehensive information available on the neurophysiology and neuroanatomy of this organism has enabled significant advances in such areas as the study of the neural basis of behavior, the development of adult-born neurons in the central nervous system and their involvement in the regeneration of nervous tissue, as well as brain aging and senescence. Despite substantial scientific interest in this species, no genomic resources are currently available.

**Results:**

Here, we report the *de novo* assembly and annotation of the *A. leptorhynchus* transcriptome. After evaluating several trimming and transcript reconstruction strategies, *de novo* assembly using Trinity uncovered 42,459 unique contigs containing at least a partial protein-coding sequence based on alignment to a reference set of known Actinopterygii sequences. As many as 11,847 of these contigs contained full or near-full length protein sequences, providing broad coverage of the proteome. A variety of non-coding RNA sequences were also identified and annotated, including conserved long intergenic non-coding RNA and other long non-coding RNA observed previously to be expressed in adult zebrafish (*Danio rerio*) brain, as well as a variety of miRNA, snRNA, and snoRNA. Shotgun proteomics confirmed translation of open reading frames from over 2,000 transcripts, including alternative splice variants. Assignment of tandem mass spectra was greatly improved by use of the assembly compared to databases of sequences from closely related organisms. The assembly and raw reads have been deposited at DDBJ/EMBL/GenBank under the accession number GBKR00000000. Tandem mass spectrometry data is available via ProteomeXchange with identifier PXD001285.

**Conclusions:**

Presented here is the first release of an annotated *de novo* transcriptome assembly from *Apteronotus leptorhynchus*, providing a broad overview of RNA expressed in central nervous system tissue. The assembly, which includes substantial coverage of a wide variety of both protein coding and non-coding transcripts, will allow the development of better tools to understand the mechanisms underlying unique characteristics of the knifefish model system, such as their tremendous regenerative capacity and negligible brain senescence.

**Electronic supplementary material:**

The online version of this article (doi:10.1186/s12864-015-1354-2) contains supplementary material, which is available to authorized users.

## Background

The brown ghost knifefish (*Apteronotus leptorhynchus*) is a weakly electric teleost fish belonging to the taxonomic order Gymnotiformes. This species has been widely studied over the past several decades as a model system in a variety of disciplines within biology and neuroscience, with particular focus on the ionic and neuromodulatory regulation of neural oscillations [1-6], neural control of communication via electric signals [7,8], and central nervous system (CNS) regeneration [9,10].

Most research involving this species has addressed diverse aspects of their nervous system. As all other species of the family Apteronotidae, *A. leptorhynchus* generates electric discharges using a neurogenic electric organ, formed by modified axonal terminals of spinal motoneurons [11]. The electric organ discharge is used for orientation and object detection in close vicinity of the fish [12,13], and for communication with conspecifics [7,14-19]. Knifefish are able to sense both their own electric discharges and electric signals of other biological and non-biological sources through electroreceptors distributed on the skin [8,20,21]. The neural structures involved in the processing of behaviorally relevant electrosensory information, and in the motor control of the electric organ discharges, are among the best characterized brain and spinal cord systems of any non-mammalian vertebrate, thus establishing *A. leptorhynchus* as a significant model of neuroethology [22].

These neuroethological investigations have yielded an extensive body of information on the structure and function of the CNS of *A. leptorhynchus*, including the first neuroanatomical atlas of the brain of any teleost species [23]. This knowledge base has, in turn, encouraged the use of the brown ghost knifefish as a model organism in several other biological disciplines, including developmental neurobiology and regenerative biology. For example, the availability of a brain atlas enabled the first comprehensive mapping of adult-born cells in the whole brain of any vertebrate species [24], establishing *A. leptorhynchus* as a major teleostean model system for the study of adult neurogenesis [9,10,25,26], and informing similar mappings of the stem cell niches in the adult brain of zebrafish (*Danio rerio*) [27] and the Mozambique tilapia (*Oreochromis mossambicus*) [28]. Utilization of this model has provided substantial insight into the cellular mechanisms that underlie the generation, migration, and differentiation of adult-born cells in various regions of the CNS [29-33]. Recently, *A. leptorhynchus* has been introduced to the area of aging research as the first vertebrate model system exhibiting negligible brain senescence [34].

Like many other teleost fish studied thus far, apteronotids have a remarkable capacity for regeneration after CNS injury, a property which has been connected to the high levels of adult neurogenesis occurring throughout the life of fish [10]. Adult neurogenesis and neuroregeneration have been studied extensively both in the brain and in the spinal cord of brown ghost knifefish. Studies using proteomic analysis in conjunction with brain lesion paradigms have contributed to a better understanding of the molecular dynamics triggered by injury and subsequent regeneration [35,36].

The unique neurogenic origin of the electric organ in apteronotids greatly facilitates the correlation of structural regeneration and functional recovery after spinal cord injuries, by allowing instantaneous non-invasive monitoring of the activity of newly formed spinal electromotor neurons. Thus, the spinal cord of brown ghost knifefish has proven to be a useful model both for understanding spontaneous regeneration of the CNS and for assessing the effectiveness of experimental manipulations aimed at improving natural recovery in regeneration-competent organisms [37-40].

Despite the extensive use of apteronotids as model systems, there are currently no large-scale genomic resources for any species of this family. Several studies have used molecular cloning and sequencing to determine partial or complete sequences of less than two dozen proteins, mostly transmembrane ion channels and receptors [41-54], but also synaptic scaffold proteins [55], enzymes [56], and homeobox genes [57]. The addition of new, detailed sequence information on a large scale is required for the development of better tools to be used in the study of the CNS in knifefish.

The present investigation describes the *de novo* assembly and annotation of the *A. leptorhynchus* CNS transcriptome based on sequencing datasets derived from Illumina-based sequencing-by-synthesis. Results are presented for libraries prepared from both the brain and the spinal cord of ten adult male and female knifefish. Translation of a subset of transcript open reading frames was experimentally validated by shotgun proteomics.

## Results and discussion

**Figure 1:**
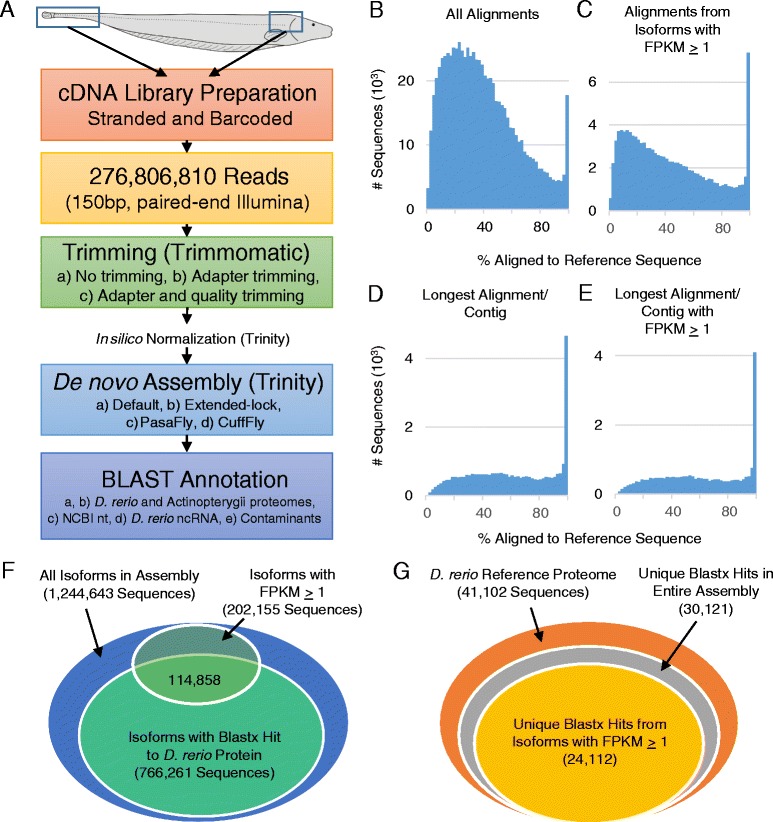
**Transcriptome assembly workflow and alignment to** ***D. rerio*** **reference proteome. A**. RNA was extracted from brain and spinal cord tissue of adult *A. leptorhynchus,* fragmented and barcoded, and strand-specific cDNA libraries were created for Illumina sequencing. Reads were trimmed using several strategies and then normalized *in silico* prior to *de novo* assembly with Trinity. Transcript reconstruction was performed using several strategies and then benchmarked using BLAST to maximize transcripts aligning to a *D. rerio* reference proteome. The best assembly was then further annotated. **B**. Out of the transcripts from the entire *A. leptorhynchus* assembly with any alignment to a *D. rerio* reference protein, most transcripts aligned to <40% of the reference *D. rerio* proteins. **C**. When filtering the assembly for transcripts with FPKM ≥ 1, the relative proportion of transcripts with less than complete alignments was reduced. **D**. When selecting the assembly transcript with the longest *D. rerio* alignment within a given contig, this greatly reduced the relative proportion of less than complete alignments. **E**. This distribution was preserved when considering only transcripts with FPKM ≥ 1. **F**. Out of the entire assembly, more than half of the transcripts had at least some alignment to a reference *D. rerio* sequence. Similarly, more than half of the sequences with FPKM ≥ 1 aligned to a *D. rerio* protein sequence. **G**. Using only transcripts with FPKM ≥ 1, nearly 60% of the reference *D. rerio* proteome had at least one assembly transcript with an alignment (24,112 sequences), which represented approximately 80% of the *D. rerio* sequences that were hit by the entire assembly (30,121 unique sequences).

Given the lack of a reference *A. leptorhynchus* genome, we adopted a *de novo* assembly and annotation strategy to evaluate assembly quality and interpret results (Figure 1A). Since complete genome sequences are available for a number of fish species, this existing information can be used to examine the quality of the presented *de novo* assembly, and to annotate it. Such genomic information is currently available for two species of pufferfish, the Japanese pufferfish (*Takifugu* [*Fugu*] *rubripes*), the first vertebrate genome to be released after the human one [58], and the spotted green pufferfish (*Tetraodon nigroviridis*) [59]; as well as for the medaka (*Oryzias latipes*) [60]; the Atlantic cod (*Gadus morhua*) [61]; the three-spined stickleback (*Gasterosteus aculeatus*) [62]; the zebrafish (*D. rerio*) [63]; the Southern platyfish (*Xiphophorus maculatus*) [64]; and, recently, the coelacanth (*Latimeria chalumnae*) [65]. Out of all teleost fish with complete genomes available, the only species that belongs to the same phylogenetic superorder (Ostariophysi) and subdivision (Otocephala) as *A. leptorhynchus*, is *D. rerio* [66,67]. Similarly, *D. rerio* is the species most closely related to *A. leptorhynchus*, according to established phylogenetics, for which a complete reference proteome sequence set is available. We therefore preferentially used the genomic and proteomic information available from *D. rerio* as a reference for sequence comparison and for *de novo* transcriptome annotation in *A. leptorhynchus*. This approach further allowed us to leverage the power of several bioinformatics tools often only available for the core model species.

### *De novo* transcriptome assembly

Strand-specific cDNA libraries were prepared from CNS tissues (brain and spinal cord) of 10 adult male and female *A. leptorhynchus*. Final raw read counts included 24,164,311, 150 bp paired-end reads passing filter on an Illumina MiSeq platform, and 252,642,499 150 bp paired-end reads passing filter obtained using an Illumina HiSeq 2500 platform. These reads were combined and then assembled using Trinity [68]. After optimizing parameters for trimming and transcript reconstruction (see below), an assembly with ~300,000 unique contigs, including ~1.2 M total isoforms, was produced. Of these, 42,459 contigs contained at least a partial protein-coding sequence based on alignment to a reference set of known Actinopterygii protein sequences. As many as 11,847 of these contigs contained full or near-full length (≥80%) unique protein sequences (based on significant sense alignments to the Actinopterygii reference set, described below), thus providing broad coverage of the *A. leptorhynchus* proteome. In general, we used BLAST alignment of transcripts (or translations of predicted ORFs from transcripts) to reference protein sets as a means of assessing coding transcript completeness. Transcripts with ≥ 80% sequence coverage (i.e. a significant alignment between a transcript sequence from our assembly and a target protein sequence, where the alignment covers at least 80% of the target protein sequence) are thus considered “full or near-full length”.

### Trimming optimization

As part of optimizing our *de novo* assembly, we compared the effect of read trimming on *de novo* transcriptome assembly. Read trimming strategy affects assembly quality and can impact downstream analysis, with the best tradeoff between read loss and dataset quality dependent upon the dataset itself as well as research goals [69]. For trimming reads prior to assembly, we evaluated no trimming, “soft” trimming, where 3’ adapter sequences present from insert read-through during sequencing were removed as well as leading and trailing bases that were uncalled (“N” bases) or with low quality (below 3), and “hard” trimming, where, in addition to the “soft” trimming criteria, a sliding window was used to eliminate bases that fall below a threshold quality (4-base wide sliding window, cutting when the average quality per base dropped below 15 [70,71]). In either of the trimmed read sets, trimmed sequences shorter than 35 bp were also removed before further analysis, as well as reads that became unpaired because of this. Prior to assembly, these trimmed read sets were pooled and normalized using the *in silico* normalization script packaged with Trinity, in order to remove highly redundant sequences and reduce assembly computational time. The trimmed, normalized read sets were then assembled with Trinity using the default settings for strand-specific, paired-end read sets, including a 200 bp minimum transcript length (additional parameters for transcript reconstruction were considered and are described below). Trinity produces transcripts that are assigned to ‘genes’ (previously referred to in Trinity as “components”), with each gene set potentially corresponding to multiple transcripts derived from the same genomic loci (i.e. transcripts within the same assembly gene are potential alternatively spliced variants). Here, we refer to unique Trinity “components” as “contigs”, where a single contig can include a set of multiple sequence variants.

In order to compare quality of assembly methods and permit further optimization, we BLASTx searched (E-value cut-off = 10^−5^) a *D. rerio* reference proteome (41,112 sequences) obtained from UniProtKB, which contained non-redundant sequences from both SwissProt and Trembl. Relative to the total number of protein sequences in this *D. rerio* reference proteome set, similar coverage was achieved regardless of trimming strategy, although the “soft” trimming strategy provided a minor advantage over “hard” trimming, recovering 2.44% more transcripts with at least 80% sequence coverage (12,446 sequences, including antisense alignments, for soft trimming compared with 12,149 sequences for hard trimming) (Additional file 1: Figure S1A). It has been reported that while the aggressive quality-based trimming strategy is common, a gentler strategy can favor increasing sensitivity of transcript reconstruction during *de novo* assembly [72].

Regardless of trimming strategy, each raw assembly was considerably large, with ~300,000 contigs and ~1.2 M total isoforms per assembly. Whereas these values were similar to the numbers observed using comparable methods [73,74], they are larger than more reasonable estimates of loci achieved with other datasets [75]. This may be due to incomplete contiguous transcript reconstruction of low-expressed transcripts, resulting in multiple (non-overlapping) contigs per locus, as well as contamination and transcriptional noise. We attempted to determine whether the size of the assembly could be further limited, while retaining coverage of the *D. rerio* proteome, by evaluating different strategies for transcript reconstruction.

### Transcript reconstruction optimization

With the goal of minimizing excessive alternative transcript reconstruction, we evaluated several reconstruction methods in Trinity. Aside from the default Trinity transcript reconstruction method, Butterfly [76], we considered Butterfly in “extended lock” mode, which favors more conservative transcript reconstruction resulting in fewer isoforms. In addition, CuffFly, an implementation of the Cufflinks assembly algorithm [74,77] that finds the minimum number of isoforms that capture the variation in the reads, and PasaFly, an implementation of the PASA (Program to Assemble Spliced Alignments) assembly algorithm [78] adapted for Butterfly transcript graphs, were also evaluated, as these methods generally tend to produce even more conservative isoform reconstructions. While the alternative transcript reconstruction methods reduced the overall number of transcripts in the assembly, they produced similar number of overall contigs (Additional file 1: Figure S1B). Despite similar number of contigs overall, the default (Butterfly) transcript reconstruction did provide some enhanced sensitivity, with 7.98% more sequences aligned than the next best reconstruction method (12,446 sequences, including antisense alignments, covered with Butterfly compared with 11,526 with PasaFly) (Additional file 1: Figure S1C).

Overall, while many contigs remained unassigned at this initial level of annotation, the identification of contigs with sense alignments covering ≥80% of 9901 *D. rerio* protein sequences indicated that the assembly was of substantial quality (Additional file 2: Table S1) despite being limited to libraries prepared from only the CNS of *A. leptorhynchus*. The large number of assembly contigs with only partial alignments suggested that higher coverage could be achieved with additional libraries from other tissues.

### Full sequence coverage enrichment amongst highly expressed transcripts

From the assembly produced by the least stringent but most sensitive Butterfly method, the number of isoforms per contig followed a power law, with many contigs having few isoforms and a few contigs having many (100–1000) isoforms (Additional file 1: Figure S1D). However, after filtering for transcripts with number of fragments per kilobase of transcript per million mapped reads (FPKM) ≥ 1, the approximate equivalent of 1 transcript per cell [79], the number of transcripts per contig was reduced to a log distribution, where even though many contigs had multiple isoforms, the number of isoforms was on the order of 10’s (Additional file 1: Figure S1E). In terms of transcript length, the overall assembly had a N50 of 2539 bp (median transcript length = 1219 bp, average length = 1606 bp, total assembled bases = ~2.0Gb, Additional file 1: Figure S1F). However, when considering only the longest transcripts per assembly contig, the distribution shifted to an N50 of 940 bp (median transcript length = 377 bp, average length = 661 bp, total assembled bases = ~230 Mb, Additional file 1: Figure S1G), suggesting that the larger N50 calculated from the entire assembly (including every isoform per contig) was due to longer contigs having many long isoforms assembled by Trinity. After filtering for transcripts with FPKM ≥ 1, the N50 was 2093 bp (median transcript length = 1140 bp, average length = 1508 bp, total assembled bases = ~303 Mb Additional file 1: Figure S1H), with a more consistent (i.e. less biased) N50 of 1995 bp (median transcript length = 907 bp, average length = 1330 bp, total assembled bases = ~110 Mb) after considering only the longest transcript per contig (Additional file 1: Figure S1I). Thus, when considering only contigs with isoforms that had an FPKM ≥ 1, the overall distribution of transcript size was improved.

Out of all 766,261 transcripts from the assembly (including variant isoforms belonging to the same contig) with an alignment to a *D. rerio* reference protein, most transcripts aligned to <40% of the respective protein (Figure 1B). When filtering the assembly for transcripts with FPKM ≥ 1, the relative proportion of transcripts with less than complete alignments was reduced (Figure 1C). When selecting only the longest aligned transcript per contig, the relative proportion of shorter alignments was reduced (Figure 1D), suggesting many assembly transcripts within a contig group contained partial open reading frames (ORFs). This distribution was preserved when considering only the longest aligned transcript per contig that also had an FPKM ≥ 1 (Figure 1E). Out of the entire assembly, more than half of the transcripts had at least some alignment to a reference *D. rerio* sequence. Likewise, more than half of the sequences with FPKM ≥ 1 aligned to a *D. rerio* protein sequence (Figure 1F). Using only transcripts with FPKM ≥ 1, nearly 60% of the reference *D. rerio* proteome had at least one assembly transcript with an alignment (24,112 sequences), which represented ~80% of the *D. rerio* sequences that were hit by the entire assembly (30,121 unique sequences, Figure 1G).

### Enrichment of sequences aligning to closely related fish species

*D. rerio* has become one of the dominant model fish species in developmental biology, and leveraging the high quality annotation developed for this fish species makes it an attractive dataset to build from in annotating the *A. leptorhynchus* transcriptome. However, as described earlier, genomic studies of several fish species exist, leading to several “complete” reference proteomes being established. To assess the relative applicability of various well-characterized fish species’ sequence data, we aligned the assembly to a well-annotated proteome sequence set consisting of seven species, including *D. rerio,* the Nile tilapia (*Oreochromis niloticus*), *X. maculatus*, *G. aculeatus*, *O. latipes*, *T. rubripes*, and *T. nigroviridis*. While the overall number of transcripts with significant alignments increased in this expanded sequence set, the majority of highest identity protein-coding sequences in the *A. leptorhynchus* transcriptome were found to align to sequences from *D. rerio* (Table 1), consistent with known phylogenetic relations between these fish species [67]. In total, 11,847 contigs had sense alignments that covered ≥ 80% of an Actinopterygii protein sequence (Additional file 3: Table S2).

**Table 1:** **Coverage of proteins for Actinopterygii species by relative number of entries in reference sets**

**Binomial name**	**Common name**	**Hits/Total (% of Total/% Relative)**
*Danio rerio*	Zebrafish	7880/41102 (66.5%/56.1%)
*Oreochromis niloticus*	Nile tilapia	1257/26753 (10.6%/13.7%)
*Xiphophorus maculatus*	Southern platyfish	654/20451 (5.5%/9.4%)
*Gasterosteus aculeatus*	Three-spined stickleback	607/27248 (5.1%/6.5%)
*Oryzias latipes*	Japanese rice fish	525/24637 (4.4%/6.2%)
*Takifugu rubripes* (*Fugu rubripes*)	Japanese pufferfish	549/47867 (4.6%/3.4%)
*Tetraodon nigroviridis*	Spotted green pufferfish	375/23073 (3.2%/4.8%)
All species		11847/211131 sequences

“% of Total” is percent of sequences out of total number of sequences across all 7 reference protein sequence sets. “% Relative” is percent of sequences normalized by the proportion of sequences from that species out of the 7 reference protein sequence sets examined. Only alignments that covered ≥ 80% of the reference protein are included in this analysis.

**Figure 2:**
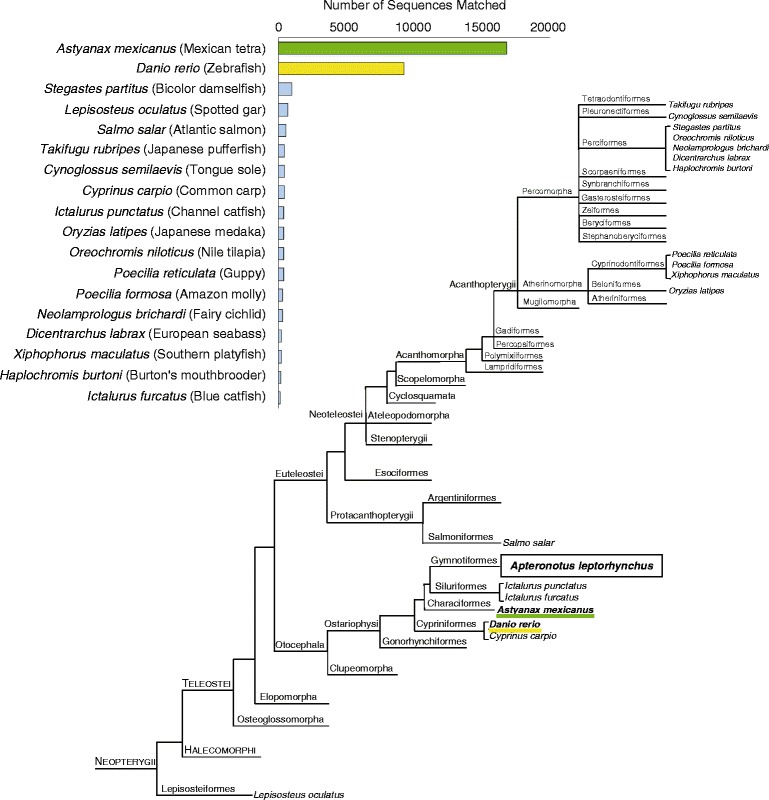
**Taxonomic classification of the** ***A. leptorhynchus*** **transcriptome assembly.** The bar chart shows the top results of the BLASTn sequence search against the entire NCBI nt sequence set (E-value cut-off = 10^−5^). Hits from assembly contigs with multiple transcripts were counted only once, using the highest scoring (bit score) transcript per assembly gene. Results were qualitatively similar when considering all transcripts individually (not shown). Note that all the top 18 hits are species of fish, with the best represented species being the characiform *A. mexicanus* (green), followed by the cypriniform *D. rerio* (yellow). The cladogram of subclass Neopterygii of the class Actinopterygii shows the phylogenetic relationships between the species (in *italics*) represented among the top BLASTn hits. The largest number of matched sequences was observed in species belonging to closely related orders (*underlined*), with the rest of the hits including a member of the order Lepisosteiformes, and numerous species in the well-represented superorder Acanthopterygii. (Cladogram compiled from literature, after: [66,67,82]).

To investigate the quality of the *A. leptorhynchus* assembly by alignment to a broader set of transcript sequences, the assembly sequences were BLASTn searched against the entire NCBI nt sequence set (E-value cut-off = 10^−5^). Sorting the BLASTn results by species showed an overwhelming representation of fishes, with the best represented species being the characiform *Astyanax mexicanus* (Mexican tetra, blind cave fish), followed by the well-characterized cypriniform *D. rerio* (Figure 2). This finding is not surprising given that these two species belong to the same taxonomic superorder, Ostariophysi. On the other hand, one would have expected more hits to the *A. leptorhynchus* assembly among two other ostariophysans, the siluriforms *Ictalurus punctatus* (channel catfish) and *Ictalurus furcatus* (blue catfish). However, this result is likely influenced by the relative abundance of the high-quality sequences available for both *D. rerio* and *A. mexicanus* [80,81].

### Transcriptome coverage assessment and enrichment analysis

**Figure 3:**
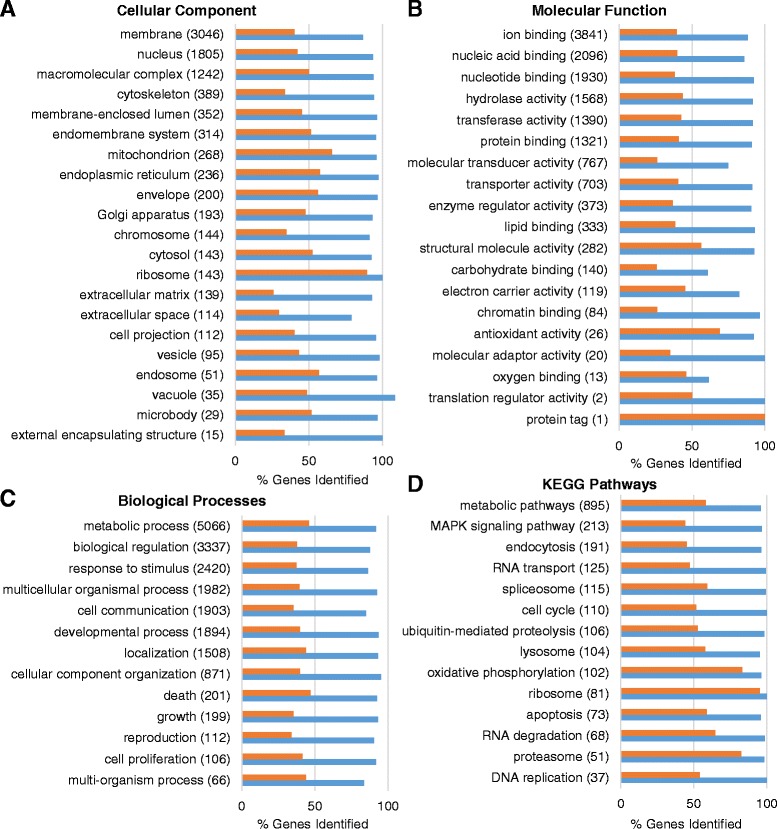
**Coverage of genes by GO-slim categories.** **A**-**C**. Percent coverage of GO-slim categories, divided by GO domains, relative to their size based on the reference *D. rerio* proteome dataset. The number of *D. rerio* genes in the reference proteome that are found in each GO category are shown in parentheses adjacent to the category name. Blue bars represent the percent of unique genes in that category with at least one *A. leptorhynchus* assembly transcript best aligned to the protein product corresponding to that gene. Orange bars show the percent of genes with an alignment from the assembly that covered at least 80% of the corresponding *D. rerio* protein sequence. **D**. Example KEGG pathways and their coverage relative to the *D. rerio* reference set.

To provide a detailed view of the coverage of the assembly in terms of diversity of protein-coding transcripts assembled, we examined the percent of genes covered by Gene Ontology (GO) categories based upon existing annotation from *D. rerio* (using the corresponding gene names for proteins in the *D. rerio* reference protein sequence set*)*. Using a generic set of GO-slim categories, we evaluated both the set of *D. rerio* protein sequences that were covered at least 80% by a sense-aligned *A. leptorhynchus* assembly sequence, as well as the set of *D. rerio* proteins that had any significant alignment to *A. leptorhynchus* assembly sequences (Figure 3A-C). While the set of *D. rerio* genes with at least partial alignments indicates that over 90% of genes in any given GO class have been identified, it remains possible that some of these incomplete alignments could be spurious or misassembled artifacts. Thus, the coverage of the more fully resolved genes can be considered a more accurate read-out of the *A. leptorhynchus* assembly coverage. Overall transcriptome coverage was balanced across major structural and functional categories. Notably, in terms of cellular components, the ribosome had the highest percentage (90%) of transcripts that were full or near-full length. This was also observed when looking at the percentage of contigs covered in major pathways from the Kyoto Encyclopedia of Genes and Genomes (KEGG) database [83,84] (Figure 3D).

The moderate-to-low coverage of many genes can be partially attributed to tissue/life stage-specific and/or weakly expressed gene transcripts. To test this hypothesis, we used the Zebrafish Expression Ontology of Gene Sets (ZEOGS), a tool that determines which anatomical structures are overrepresented in a given input gene set [85]. Indeed, when examining enrichment of adult *D. rerio* genes that had over 80% sequence coverage by *A. leptorhynchus* assembly transcripts, a variety of nervous system-related terms, including central nervous system, spinal cord, optic tectum, epiphysis, etc., were significantly enriched (Benjamini-Hochberg adjusted *p* < 0.05, Table 2). By contrast, the set of *D. rerio* sequences with low-to-no sequence coverage in the *A. leptorhynchus* assembly were significantly enriched for various other anatomical terms, including components of the reproductive and skeletal systems as well as the heart. This method of classification could be used in future analyses to help prioritize tissues for library preparation and sequencing to further expand transcriptome coverage.

**Table 2:** **Anatomical enrichment of genes with high sequence coverage compared with genes having lower to no coverage**

**Significantly enriched anatomical terms sequences with high (≥80%) coverage**		**Significantly enriched anatomical terms sequences with < 80% or no coverage**	
Optic tectum	0.00004	Ovary	0.03556
Epiphysis	0.00007	Testis	0.04287
Eye	0.00032	Olfactory rosette	0.04300
Central nervous system	0.00038	Cardiac ventricle	0.04821
Retina	0.00147		
Liver	0.00167		
Blood	0.00507		
Neuron	0.00548		
Cranial nerve II	0.01609		
Cranial ganglion	0.01633		
Spinal cord	0.01833		
Cardiovascular system	0.01889		
Macrophage	0.02454		
Occipital lateral line neuromast	0.02617		
Myotome	0.03472		
Ventral thalamus	0.04871		

Analysis was performed using ZEOGS, comparing enrichment of *D. rerio* genes with ≥ 80% sequence coverage (left column) compared with the list of *D. rerio* genes with < 80% or no sequence coverage (right column). Only anatomical terms that were significantly enriched (Benjamini-Hochberg adjusted α = 0.05) from either list are shown along with their Benjamini-Hochberg adjusted *p*-values.

**Figure 4:**
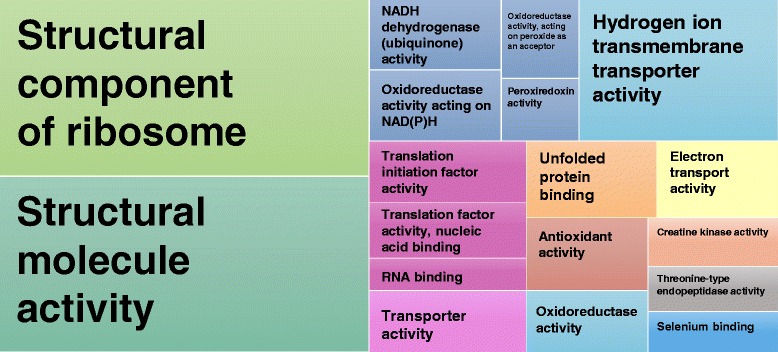
**Gene Ontology treemap based on enrichment of most highly expressed transcripts.** Assembly contigs that aligned to ≥80% of a reference *D. rerio* protein were ranked by FPKM, and the GO classification for the corresponding *D. rerio* gene were used to determine enrichment of transcripts at the top of the list using GOrilla based on the minimum hypergeometric scoring method. GOrilla analysis showed significantly enriched GO categories that were found on average in the top 9.6% of genes (±4.8% S.D.) using minimum hypergeometric scoring. Redundant GO terms were filtered with the GO Trimming tool and then visualized with REViGO. The box size correlates to the –log10 p-value of the GO-term enrichment. Boxes with the same color are grouped by semantic similarity (SimRel, similarity = 0.7).

To assess which molecular categories were most highly expressed among those transcripts of the *A. leptorhynchus* assembly that aligned to ≥80% of a reference *D. rerio* protein, assembly transcripts were first ranked by FPKM, and the GO classification for the corresponding *D. rerio* genes was used to determine enrichment of the most highly expressed transcripts using GOrilla [86]. GOrilla uses the variable enrichment thresholding method of minimum hypergeometric scoring to determine GO enrichment from ranked gene lists, with significantly enriched GO categories found on average in the top 9.6% of genes (4.8% S.D.). When considering the relative expression levels of transcripts with full or near-fully resolved ORFs, structural components of the ribosome and factors involved in translation were found to be most prominent (Figure 4, Additional file 4: Table S3), consistent with the observation that the ribosome was the cellular component that had the highest percentage of well-resolved genes. Expression of transport-related genes was also observed to be enriched, which is consistent with a similar analysis performed after the *de novo* assembly of the black-faced blenny (*Tripterygion delaisi*) transcriptome [73].

### Extension of previously sequenced knifefish transcripts

**Figure 5:**
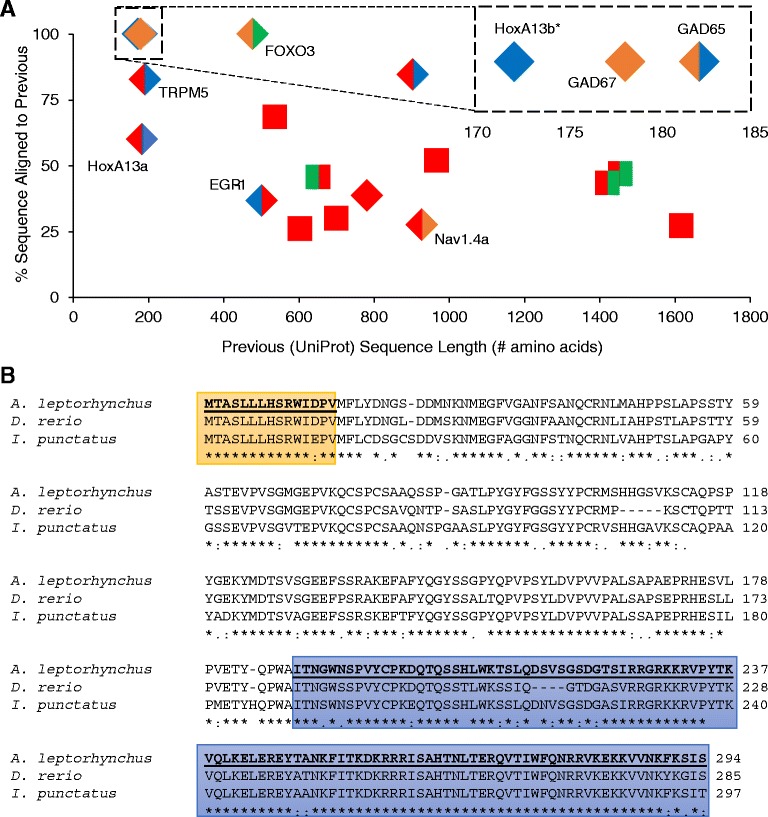
**Identification and extension of previous** ***A. leptorhynchus*** **sequences. A**. All 18 *A. leptorhynchus* protein sequences deposited in UniProt plotted individually to illustrate the previously determined sequence length compared with the percent aligned to an assembly sequence. UniProt sequences annotated as fragments are denoted with diamonds. The left and right halves of the markers denote the completeness of the N- and C- termini, respectively, of the best aligned sequence from our final assembly (green = our terminus aligned with the previously complete terminus, red = our sequence is missing terminus sequence, orange = our sequence has extended sequence compared with the previous sequence, but it is likely still not complete, blue = our terminus extends the sequence to a likely terminal residue). Extended sequences are labeled. Inset shows sequences clustered around 172–182 amino acids long that all had 100% coverage. **B**. HoxA13b (asterixed in A) was extended to completion based on identification of a stop codon, as well as a start codon that aligned to the start of sequences from *D. rerio* and *I. punctatus* (extended termini are highlighted in colored boxes). Alignment performed with ClustalW2: * (asterisk) = fully conserved residue; : (colon) = conservation between groups of strongly similar properties (>0.5, Gonnet PAM 250 matrix); . (period) = conservation between groups of weakly similar properties (≤0.5).

Previous cloning and characterization of *A. leptorhynchus* genes included characterization of N-methyl-D-aspartate receptors [43-45,50], HoxA paralogs [57], early growth response-1 (egr-1) [87], voltage-gated sodium and potassium channels [42,46,49], Forkhead box transcription factors [88], TRP channels [47], and glutamate decarboxylases [56]. All 18 *A. leptorhynchus* protein sequences deposited in UniProt had at least a partial sequence in the assembly (Figure 5A). Of note, the sequence of HoxA13b was determined to be extended to completion here based on identification of a stop codon, as well as a start codon that aligned to the start of multiple homologous sequences from related species including *D. rerio* and the channel catfish (*I. punctatus*), a member of the order Siluriformes more closely related phylogenetically to *A. leptorhynchus* (Figure 5B).

### Identification of non-coding RNA (ncRNA) expression and antisense transcripts

The importance and complexity of ncRNAs have attracted increasing attention over the last decade. Numerous classes of ncRNA are now known to be important modulators of gene expression, involved in the regulation of a wide variety of physiological and developmental processes [89,90]. In the CNS, ncRNAs are tightly regulated, and appear to play prominent roles in developmental processes such as neurogenesis and neuronal differentiation [89-91], which continue throughout adult life in *A. leptorhynchus* [9,10,26,92].

To investigate ncRNA present in the *A. leptorhynchus* assembly, we compared assembly transcripts to a reference *D. rerio* ncRNA dataset (Danio_rerio.Zv9.75.ncrna, 8319 sequences) with BLASTn (Table 3, Additional file 5: Table S4), focusing our analysis on several major ncRNA classes including ribosomal RNA (rRNA), microRNA (miRNA), small nuclear RNA (snRNA), small nucleolar RNA (snoRNA), long non-coding RNA (lncRNA), and antisense RNA to protein-coding genes.

**Table 3:** **Summary of annotated transcripts/contigs by RNA types**

**Categories**	**# Transcripts (including contig isoforms)**	**# Contigs**
**Protein sequences**		
Sense BlastX (*D. rerio*)	748,404	40,451
Sense BlastX (Actinopterygii)	759,947	42,459
**rRNA**		
rRNA (identified by RNAmmer)	59	4
rRNA (from *D. rerio* reference)	2	2
Mitochondrial rRNA (from *D. rerio* reference)	4	2
Other ncRNA*		
snoRNA	211	17
miRNA	340	84
snRNA	116	12
lincRNA	419	54
SRP RNA	1	1
Antisense	75	27
lncRNA (Kaushik et al.)	111	15

Note that some transcripts were found to align to multiple categories, such as the case of transcripts containing protein-coding ORFs as well as a pri-miRNA sequence. *ncRNA was identified from *D. rerio* reference (ftp://ftp.ensembl.org/pub/release-75/fasta/danio_rerio/ncrna/Danio_rerio.Zv9.75.ncrna.fa.gz) except for rRNA identified with RNAmmer and lncRNA from [93].

While our library preparation protocol was designed to limit the presence of rRNA, two contigs were found to align to *D. rerio* 5S and 5.8S rRNA, with two additional contigs corresponding to mitochondrial rRNA. Using an additional rRNA prediction tool, RNAmmer [94], four additional contigs were designated as 8 s rRNA (comp134303, comp138865, comp150483, and comp193485).

A total of 84 of the *A. leptorhynchus* assembly contigs were aligned to 81 miRNA transcripts from *D. rerio*. Out of these, 54 *A. leptorhynchus* assembly transcripts aligned to ≥80% of the corresponding *D. rerio* miRNA. Many of these alignments to miRNA sequences were included into longer assembly transcripts, which had portions that aligned to *D. rerio* protein sequences, suggesting that these transcripts likely included precursor miRNAs (pri-miRNAs), transcribed in conjunction with adjacent protein coding sequences [89].

Thirteen putative snRNA were identified, including all five snRNA components of the spliceosome (U1, U2, U4, U5, and U6), as well as U11 and U12 minor spliceosomal RNA. Also detected was 7SK small nuclear RNA (rn7sk), the RNA component of the 7SK snRNP involved in the control of transcription elongation and in the regulation of pre-mRNA splicing [95]. The RNA component of the signal recognition particle (SRP), a universally conserved ribonucleoprotein that targets specific proteins to the endoplasmic reticulum in eukaryotes [96,97], was also detected, with 95.1% identity across 96.6% of the corresponding *D. rerio* transcript.

Seventeen transcripts aligned to snoRNA, a family of RNA which guide chemical modification (methylation and pseudouridylation) of nascent rRNA to generate mature rRNA [90]. Amongst the snoRNA identified were 13 methylation-associated C/D-box class snoRNA and 4 pseudouridylation-associated H/ACA-box class snoRNA.

A total of 54 sequences of the *A. leptorhynchus* transcriptome showed significant alignment to known *D. rerio* long intergenic non-coding RNA (lincRNA), including two sequences that covered >90% of the corresponding *D. rerio* lincRNA. While sequence coverage was generally low, alignments to >1000 nucleotides with close to 80% identity were found.

To further investigate the presence and conservation of lncRNA expressed in adult CNS tissues of *A. leptorhynchus*, the assembly was searched against an additional set of lncRNAs expressed in adult *D. rerio*, including many with specific expression in the brain. LncRNAs have been shown to be expressed with high tissue specificity, in particular in the brain [98], where they have been associated with the control of neuronal diversification and specification [91]. Kaushik et al. reported a set of 419 novel lncRNAs, including 47 specifically expressed in adult *D. rerio* brain when compared with heart, liver, muscle, and blood [93]. Amongst these, we found evidence for at least one conserved lncRNA expressed exclusively in the brain (lncBr_002), as well as 10 additional lncRNAs found in adult *D. rerio* brain and other tissues examined, and finally 4 lncRNAs that were not observed in the brain previously, but were found in blood. It is possible that these transcripts could be novel, functional lncRNA. However, further investigation will be required to exclude the possibility of contamination from remaining blood vessels in the isolated brain tissue.

Antisense transcription contributes to the complexity of expression dynamics, and while antisense transcripts can play roles in regulating translation and splicing, separating functional antisense transcripts from transcriptional noise remains a challenge. Out of the 682 antisense sequences in the available *D. rerio* ncRNA reference set, only 25 had significant alignments (e-value < 10^−10^) to transcripts from 27 contigs in the *A. leptorhynchus* assembly, with significant alignments ranging from 46 bp to 930 bp. Expression of thousands of antisense transcripts was found to be conserved across humans, mice, and rats, although to a lesser degree than protein-coding genes [99]. The relatively low coverage of antisense transcripts from *D. rerio* may be due to lack of sufficient sequencing depth or to the restriction of our analysis to RNA expressed in adult CNS tissue.

### Detection of protein-coding ORFs and putative protein-coding gene duplications

To determine the overall protein-coding potential of transcripts in the *A. leptorhynchus* assembly, regardless of their alignment to known Actinopterygii protein sequences, protein-coding ORFs were predicted from the assembly using TransDecoder [68], both with and without biasing “best” ORF calling towards ORFs with a recognizable domain in the Pfam protein families database [100]. When limiting the set of ORFs from each search to only those comprising at least 100 amino acids, the resulting distributions of ORF lengths were similar overall, regardless of whether or not Pfam domains were used for guiding ORF selection (Additional file 6: Figure S2A-B). Similarly, filtering for transcripts with FPKM ≥ 1 had little effect on ORF size distribution without Pfam (Additional file 6: Figure S2C-D), suggesting that resolution of the distribution of ORF lengths of 100 amino acids or greater was independent of expression levels. BLAST aligning ORFs with or without guidance from Pfam against the *D. rerio* reference sequence demonstrated that the Pfam option did slightly increase identification of ORFs with conserved protein coding sequences (Additional file 6: Figure S2E-F). Out of the approximately 202,000 *A. leptorhynchus* transcripts with FPKM ≥ 1, 87,945 transcripts with ORFs comprising at least 100 amino acids (as found with Pfam) aligned to *D. rerio* protein sequences. Transcripts with FPKM values ≥ 1 covered a total of 21,638 unique *D. rerio* proteins, or over 80% of the identifiable sequences (Additional file 6: Figure S2G). When transcripts with FPKM values < 1 were included, this coverage increased to 26,588 unique *D. rerio* sequences, indicating that even transcripts expressed at low levels could still provide recognizable sequences. An additional 6,090 transcripts with FPKM ≥ 1 contained ORFs of over 100 amino acids that did not align to a *D. rerio* protein in our reference sequence set. However, after BLAST aligning these ORFs against the entire NCBI non-redundant (nr) protein sequence database, 3,505 additional sequences had significant alignments -- 3,273 of these alignments were to sequences from Teleostei species (Additional file 6: Figure S2H) showing a similar trend to the results from the nt sequence set, including 2,079 *A. mexicanus* sequences and 425 *D. rerio* sequences absent from the UniProt reference set.

**Figure 6:**
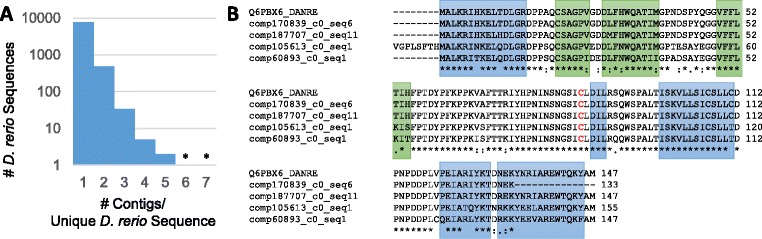
**Multiple contigs aligning to the same** ***D. rerio*** **protein sequence. A**. A histogram (log-scale) showing the number of contigs aligning to the same *D. rerio* protein sequence (with > 80% sequence coverage of the *D. rerio* sequence). While most contigs align to a single *D. rerio* sequence (7,692 sequences), 491 *D. rerio* proteins have alignments to exactly two contigs in the assembly and 34 *D. rerio* proteins have alignments to exactly three contigs (Additional file 7: Table S5). In addition, five *D. rerio* proteins have alignments to exactly 4 contigs, two *D. rerio* proteins align to five contigs, and two *D. rerio* proteins align to either 6 or 7 contigs (Table 4). **B**. Example reference *D. rerio* protein sequence, ubiquitin-conjugating enzyme E2 D2 (UBC4/5 homolog, yeast) (Q6PBX6_DANRE), which had four contigs in the assembly with alignments covering ≥80% of the Q6PBX6_DANRE sequence. Shown are the ORFs from the transcripts from each assembly contig with the longest alignment. Blue and green boxes show α-helices and β-strands, respectively, as determined from the X-ray crystal structure of the highly homologous human ubiquitin-conjugating enzyme E2 D2 (PDB ID: 2C4O). The active site cysteine is shown in red. All assembly ORFs contained a stop codon, which aligned with the C-terminal of Q6PBX6_DANRE, with the exception of comp170839_c0_seq6, which had a stop which eliminates the final helix. The ORF for comp105613_c0_seq1, as determined by TransDecoder, contained additional sequence in the N-terminal region, although this may not be translated since there was no evidence for an upstream start codon, and a methionine showed alignment in agreement with the other sequences. Alignment performed with ClustalW2: * (asterisk) = fully conserved residue; : (colon) = conservation between groups of strongly similar properties (>0.5, Gonnet PAM 250 matrix); . (period) = conservation between groups of weakly similar properties (≤0.5).

In order to identify potential *A. leptorhynchus* duplicated genes (relative to *D. rerio*), we examined instances where multiple distinct assembly contigs were aligned to the same *D. rerio* protein. Only sufficiently large contigs, with transcripts that could be aligned to at least 80% of a given *D. rerio* protein sequence, were selected. Using this threshold, we observed a 1:1 correspondence between assembly contigs and unique *D. rerio* proteins for 7,692 sequences. There were 491 instances of two contigs aligning to the same *D. rerio* protein, and 41 instances of three or more contigs aligning to the same *D. rerio* protein (Figure 6A, Additional file 7: Table S5).

One example of multiple distinct *A. leptorhynchus* assembly contigs aligning to the same *D. rerio* protein is ubiquitin-conjugating enzyme E2 D2 (UBC4/5 homolog, yeast) (Q6PBX6_DANRE, Figure 6B), in which case four *A. leptorhynchus* assembly contigs aligned to one known *D. rerio* protein. In this case, the four sequences are generally well conserved, including the cysteine active site. A more divergent example is the uncharacterized *D. rerio* protein A2BHK0, where four *A. leptorhynchus* assembly contigs had substantially different alignments at the termini (Additional file 8: Figure S3). Several examples of multiple contigs aligning to the same *D. rerio* protein sequence included proteins with multiple coding genes such as calmodulin, a Ca^2+^ binding protein involved in mediating a variety of cellular responses to variations in Ca^2+^ levels [101], which is coded for in six separate loci of the *D. rerio* genome, and seven assembly contigs of the *A. leptorhynchus* CNS (Table 4).

**Table 4:** ***D. rerio*** **proteins with ≥ 4 contigs aligning to ≥ 80% of the** ***D. rerio*** **sequence**

***D. rerio*** **reference protein (UniProt ID)**	**Protein name**	**# Contigs in assembly**	**# of** ***D.rerio*** **genes**
CALM_DANRE	Calmodulin (CaM)	7	6
E7F3G4_DANRE	Uncharacterized protein	6	1
H33_DANRE	Histone H3.3	5	4
E7FDA5_DANRE	Uncharacterized protein	5	1
A2BHK0_DANRE	Uncharacterized protein	4	1
F1R8F5_DANRE	Uncharacterized protein (fragment)	4	1
F8W544_DANRE	Phosphatidylinositol transfer protein, alpha b	4	1
Q6P2T9_DANRE	Cd63 antigen	4	1
Q6PBX6_DANRE	Ubiquitin-conjugating enzyme E2D 2 (UBC4/5 homolog, yeast)	4	1

Multiple sequence alignment of assembly sequences to phosphatidylinositol transfer protein (F8W544_DANRE) is shown in Figure 6B. Similarly, a multiple sequence alignment of assembly sequences compared to uncharacterized *D. rerio* protein (A2BHK0) is shown in Additional file 8: Figure S3.

To exclude the possibility that contigs we assigned as putative duplicated genes (relative to *D. rerio*) are the result of artifactual contig splitting by Trinity, we compared at the nucleotide level the ORFs of the 491 contigs assigned as having a single duplication (i.e. two distinct contigs with full or near-full length ORFs that align to a single *D. rerio* protein sequence). Aligning the ORFs of these pairs of contigs, we observed substantially different sequences at the nucleotide level (sequence identity = 61 ± 8%, μ ± σ), with sequence identity ranging from a maximum of 87% and minimum of 34%. Such a high dissimilarity between two ORF sequences suggests these contigs derive from different genomic loci. That both sequences produce significant alignments covering > 80% of the same target protein sequence (from *D. rerio*) suggests that these contigs are both: 1) full or near-full length; and 2) encode related protein products.

### Validation of putative protein coding regions via shotgun proteomics analysis of the *A. leptorhynchus* CNS

**Figure 7:**
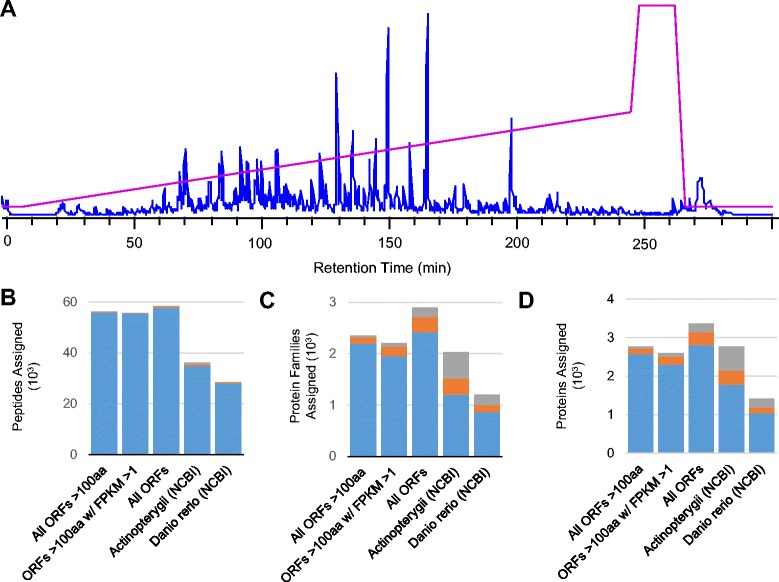
**Utility and optimization of** ***de novo*** **assembly for LC-MS/MS shotgun proteomics database searching. A**. Representative base peak chromatogram (blue) of trypsin digested *A. leptorhynchus* central nervous tissue protein extract across a 4 hour gradient (magenta). **B**-**D**. Enhancement of IDs by database by peptide ID, Mascot-assigned protein families, and individual protein at multiple false discovery rate thresholds (1% = blue bar, 2% = orange bar, 5% = grey bar).

To confirm translation of assembly ORFs, including alternative isoforms of particular contigs, and to demonstrate the usefulness of the assembly for increasing peptide assignments during tandem mass spectrometry analysis, we characterized protein extracts harvested from *A. leptorhynchus* brain and spinal cord by shotgun proteomics. Two sample preparation methods, the popular in-gel digest [102], and the recently introduced enhanced filter-aided sample preparation method [103] (eFASP, which tends to enrich membrane-bound proteins), were used. Tissue samples from both brain and spinal cord were used in order to broaden and enhance our coverage of the CNS proteome. Base peak chromatograms across a 4-hr gradient (5–40% acetonitrile) revealed a complex mixture of digest peptides (Figure 7A).

To analyze tandem mass spectrometry data, we used Mascot, which utilizes probability-based scoring in order to match tandem mass spectra to protease digest peptides predicted from a given sequence database [104]. In Mascot, limiting the reference database size, as occurs when searching only the appropriate genus and species, increases probability-based scoring and leads to a greater percentage of statistically significant identifications. Alternatively, including more sequences, for example incomplete fragments, such as those found in our assembly, or proteins from other organisms that may not have been covered in the present assembly, could increase the total number of protein IDs, but may negatively impact scoring based on adjustments due to database size. We therefore considered several approaches when preparing our assembly as a sequence database for Mascot, including using only long ORFs (at least 100 amino acids), only ORFs from transcripts with FPKM values ≥ 1, or all ORFs regardless of size, and compared these results to results obtained using available sequences of *D. rerio*, as well as other Actinopterygii sequences, from NCBI.

Notably, any ORF set derived from the *A. leptorhynchus* assembly provided the most assignments at both the peptide and protein level when compared with the results from other existing databases. The results were compared using several different false discovery rates (FDR) (Figure 7B-D). As many as 2,813 proteins were identified (from 58,106 peptide assignments) with the conservative 1% FDR from the database containing all assembly ORFs (Additional file 9: Table S6). Within Mascot-assigned “protein families”, similar protein sequences identified from a given database are grouped together. These groups can include paralogous sequences (both known and unique) and alternatively spliced variants. When comparing the Mascot protein families with annotations from BLAST results, there was good agreement between the Mascot assigned protein families and known paralogues identified from alignment with *D. rerio*. For example, the first protein family assigned by Mascot included twelve ORFs from the *A. leptorhynchus* assembly that corresponded, after BLAST searching, to multiple isoforms of alpha tubulin, including *tuba1a*, *tuba1c*, *tuba8l*, *tuba8l2*, *tuba8l3*, and *tuba8l4*. Likewise, the second protein family assigned by Mascot included 15 ORFs from the *A. leptorhynchus* assembly that all aligned to beta tubulin isoforms. The 2,813 proteins identified (FDR < 1%) from the database containing all assembly ORFs were grouped by Mascot into 2,424 protein families.

Limiting the number of sequences in the database (by either filtering by FPKM or by ORF length) did not benefit the probabilistic assignment of tandem mass spectra, and including more sequences, such as partial and low-expression sequences, was not observed to be a limiting factor. Including shorter and less abundant transcripts from the *A. leptorhynchus* assembly helped in peptide assignment. In the present study, short ORFs identified through proteomics often represented terminal fragments of transcripts that were not fully assembled (these ORFs lacked 5’ and/or 3’ ends).

**Figure 8:**
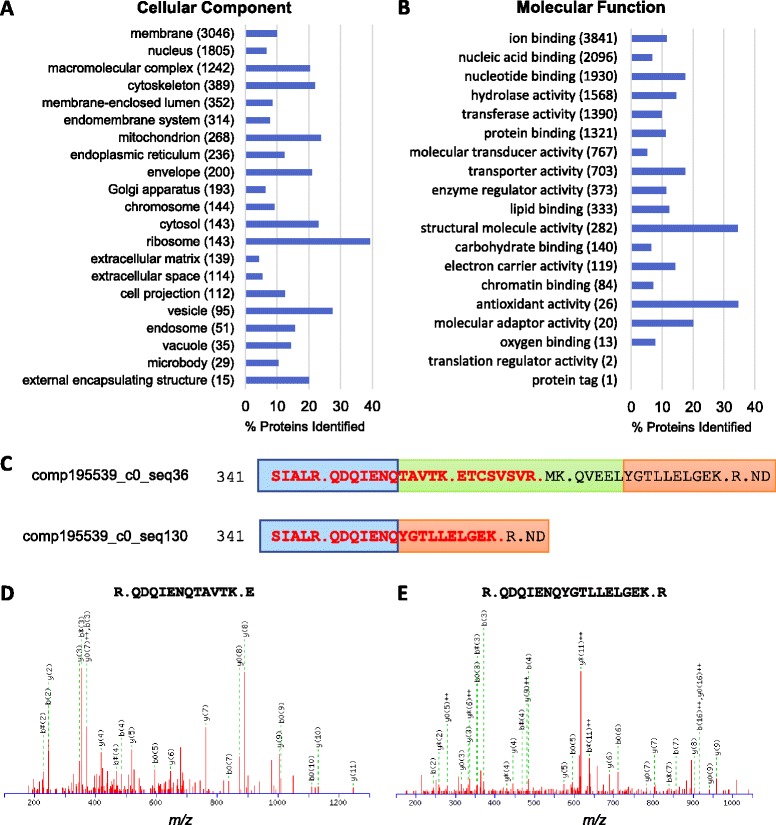
**Proteins identified by GO-slim categories and identification of splice junction peptides.** **A**, **B**. Percentage of proteins identified from (FDR < 1%, ORF database derived from TransDecoder with Pfam and any length ORF) in each GO-slim category based on number of assignable *D. rerio* genes. **C**. Region of *A. leptorhynchus* spectrin alpha 2 that includes alternatively spliced exon containing sequence TAVTKETCSVSVRMKQVEEL. Periods c-terminal to K and R indicate trypsin cleavage sites. Identified peptide sequences are red and bolded. Colored boxes emphasize exons. **D**, **E**. Annotated MS/MS spectra of splice junction peptides QDQIENQTAVTK and QDQIENQYGTKKEKGEK.

After matching the final list of identified ORFs from the *A. leptorhynchus* proteomics dataset with BLAST results from *D. rerio*, we examined the coverage of various GO categories. Consistent with the transcriptome assembly described above, the ribosome was the best-covered cellular component by proteomics, with peptides being assigned to nearly 40% of the protein constituents of the ribosome (Figure 8A). In terms of molecular function, we observed the highest coverage of structural molecules, as well as antioxidant proteins, reflecting mainly the prominence of mitochondrial antioxidant enzymes (Figure 8B).

A powerful benefit of combined transcriptomics and proteomics is the ability to confirm translation of alternatively spliced transcripts through the detection of splice junction peptides [105]. For example, within the spectrin family, which contained nine ORFs that were identified by proteomics, including *spta2*, *sptb*, *sptbn1*, *sptbn2*, there were two assembly ORFs derived from different transcript isoforms within the same contig, both aligned to *D. rerio spta2*. These two ORFs, with lengths of 1760 and 1780 amino acids, and both assigned as complete sequences based on TransDecoder, were identical except for the addition sequence TAVTKETCSVSVRMKQVEEL, present after amino acid Q352. Comparison of MS/MS sequence coverage confirmed the identification of unique splice junction peptides from each sequence (Figure 8C-E). Overall, there were 209 contigs identified in the proteomics results that had 2 or more isoforms with non-redundant ORFs identified (with two contigs each having 5 transcripts identified), although manual inspection of MS/MS for putative splice junction peptides is necessary to rule out false positives.

## Conclusions

The present study represents the first major collection of transcriptomics data for a species of the order Gymnotiformes, weakly electric fish from South and Central America with great relevance for neurobiological research. Previously, only nineteen *A. leptorhynchus* mRNA sequences were publically available. With our *de novo* assembly of the adult *A. leptorhynchus* CNS transcriptome, broad coverage of protein-coding sequences was achieved, with as many as 11,847 contigs presenting full or near-full length ORFs. Our study provides the first survey of a broad variety of ncRNA in *A. leptorhynchus*, including miRNA, snRNA, snoRNA, and other ncRNA sequences. Shotgun proteomics confirmed translation of ORFs from over 2,000 transcripts, including alternative splice variants. Our *de novo* transcriptome-to-proteome analysis contributes to the growing trend of incorporating these two techniques for enhancing shotgun proteomics identifications while confirming ORF assignment from transcriptome assembly experiments.

*A. leptorhynchus* is an important model organism in particular for the study of fundamental neurobiological aspects, such as adult neurogenesis, neuronal regeneration, and the neural basis of behavior. The availability of a targeted CNS reference transcriptome will provide novel molecular tools to explore the underlying cellular mechanisms of such phenomena. In addition, the sequence information provided here can be employed to study phylogenetic relationships and various aspects of CNS evolution in vertebrates. Future studies, using libraries from different tissues and developmental stages, will further improve the quality and applicability of the *A. leptorhynchus* assembly.

## Methods

### Animals

Brown ghost knifefish (*Apteronotus leptorhynchus*) were supplied by tropical fish importers and maintained in the laboratory as described previously [17]. A total of 10 individuals were used for sequencing, and 26 fish were used for the proteomics experiments. Both males and females were included, and all fish were adults in their second or third year of life. All animal experiments were approved by the Institutional Animal Care and Use Committee of Northeastern University. All efforts were made to reduce the number of animals used and to minimize animal suffering.

### RNA-Seq library preparation and paired-end sequencing

Total RNA was extracted from brain samples collected individually from 3 adult females, as well as pooled spinal cord samples collected from 7 male and female individuals, using an Aurum Total RNA Fatty and Fibrous Tissue Kit (Bio-Rad, Hercules, CA). RNA quality was determined by calculating the RNA Integrity Number (RIN) after performing a Eukaryote Total RNA Pico assay on a Bioanalyzer 2100 (Agilent, Santa Clara, CA). A strand-specific cDNA library for Illumina-based sequencing-by-synthesis was created using the TruSeq Stranded mRNA Sample Prep kit (Illumina, San Diego, CA). Briefly, polyadenylated mRNA was purified using poly-dT oligo-attached magnetic beads. Following purification, the mRNA was fragmented into small pieces using divalent cations under elevated temperature. The cleaved RNA fragments were reverse transcribed into first strand cDNA using reverse transcriptase and random primers. This was followed by second strand cDNA synthesis using DNA Polymerase I and RNase H in the presence of dUTP, which is incorporated into the second strand to prevent its amplification during subsequent PCR [106]. 3’ ends of double-stranded cDNA fragments were adenylated to permit ligation of barcoded adapters that allow for sorting of sequenced fragments that had been pooled prior to sequencing. The products were then purified and enriched with PCR to create the final strand-specific cDNA library. Concentration and quality of strand-specific cDNA libraries was assessed by NanoDrop (Thermo Scientific, Waltham, MA) and High Sensitivity DNA assay on a Bioanalyzer 2100, respectively. Paired-end sequencing (2 × 150 cycles) of pooled, strand-specific cDNA libraries derived from mRNA-enriched extracts was performed on MiSeq and HiSeq 2500 instruments (Illumina). After sequencing, reads were mapped to their respective samples based on ligated barcoded adapter sequences with CASAVA 1.8.2 (Illumina). Read quality was examined with FastQC and FastqScreen, which confirmed that read fidelity was maintained during sequencing and contamination from other organisms was negligible.

### *De novo* transcriptome assembly and transcript annotation

Reads were trimmed with Trimmomatic [107]. The “soft trimming” read set included reads trimmed to eliminate adapter sequences, leading and tailing bases with quality score less than 3 (including uncalled bases), and reads shorter than 35 bp after trimming. The “hard trimming” read set included reads trimmed with the “soft trimming” parameters as well as a 4-base wide sliding window, cutting when the average quality per base dropped below 15. Unpaired reads remaining after trimming were also removed in either case.

The trimmed read sets were then assembled using Trinity [68,76] (November 11, 2013 release) with default settings (which include minimum transcript length of 200 bp) for strand-specific, paired-end read sets. To reduce assembly time, trimmed reads were normalized prior to assembly using the *in silico* normalization script included with Trinity with default settings for strand-specific sequences. To evaluate the necessity of extensive alternative transcript reconstruction, additional Trinity assemblies were also performed using the extended lock feature of Butterfly. CuffFly and PasaFly were also examined as alternative transcript reconstruction methods.

To compare quality of Trinity assemblies based on trimming and transcript reconstruction strategies, sequences were blasted (BLASTx, version 2.2.28, E-value cut-off = 10^−5^) against a locally installed BLAST database containing the UniProtKB *D. rerio* complete proteome sequence set (41,102 sequences, retrieved April 2nd, 2014). The best assembly was further examined with BLASTx against two sequence sets (also retrieved April 2nd, 2014 from UniProtKB), including: an *A. leptorhynchus* protein set (18 sequences) and an Actinopterygii complete proteome set (211,131 sequences). The NCBI nt sequence set (retrieved August 17th, 2014) was used to BLASTn search the assembly. To identify ncRNA sequences, transcripts were blasted (BLASTn) against the Ensembl [108] *D. rerio* ncRNA database [109] (ftp://ftp.ensembl.org/pub/release-75/fasta/danio_rerio/ncrna/Danio_rerio.Zv9.75.ncrna.fa.gz), and an additional set of 417 lncRNA found in adult *D. rerio* [93]. RNAmmer 1.2 [94] was used to predict rRNA sequences in transcript assembly using scripts within Trinotate and HMMER 2.3.2 [110]. Vector and primer contamination was identified by VecScreen (http://www.ncbi.nlm.nih.gov/tools/vecscreen/) and contaminants were removed prior to depositing with NCBI. FPKM (fragments per kilobase of exon per million fragments mapped) of transcripts was calculated by estimating transcript abundance using RSEM (RNA-Seq by Expectation Maximization) [111], followed by TMM (trimmed-mean of *M*-values) normalization within Trinity.

### Gene ontology enrichment and KEGG pathway analysis

The WEB-based GEne SeT AnaLysis Toolkit (WebGestalt) [112,113] was used to map transcripts from blastX analysis of the *D. rerio* reference proteome to GO-slim categories and KEGG pathways. For this analysis, the corresponding *D. rerio* gene name for BLASTx-matched sequences with greater than 80% subject sequence coverage were used. For GO enrichment analysis, the same gene list was ranked by FPKM. In the case of multiple transcripts aligned to the same *D. rerio* protein, the FPKM of the highest expressed transcript corresponding to a given *D. rerio* protein was used to determine its rank in the list. The ranked list was analyzed with GOrilla [86,114] (Gene Ontology enRIchment anaLysis and visuaLizAtion tool). This process was performed similarly for proteomics results, using the *D. rerio* gene names from the BLASTp searches of the corresponding putative ORFs from TransDecoder (described below). Significant GO terms (Benjamini-Hochberg adjusted *p*-value < 0.001) were hard trimmed for redundancy using the GO Trimming tool [115]. REVIGO [116] was then used to analyze the trimmed GO IDs and corresponding *p*-values to further combine GO categories (0.5 similarity) and visualize the results with a treemap scaled by -log_10_*p*-values. For anatomical expression enrichment analysis, gene lists were analyzed with ZEOGS [85] using gene anatomical annotation from ZFIN and the adult stage filter (Benjamini-Hochberg adjusted hypergeometric *p*-value < 0.05).

### Protein extraction and sample preparation for proteomics

Brain and spinal cord tissues were isolated rapidly on ice and stored at −80°C. To increase overall coverage of identified proteins, samples were prepared using both in-gel digestion and eFASP. In-gel digestion was performed as previously described [117]. Tissue samples were homogenized for 3 min on ice in lysis buffer containing 50 mM Tris, 120 mM NaCl, 1 mM EDTA, 1% Triton X-100, 0.1% SDS, 10% glycerol (all from Fisher Scientific), pH 7.4, using a hand-held pestle homogenizer. The homogenate was placed in a rotating mixer for 40 min at 4°C, centrifuged for 10 min at 16,000×g, and the supernatant was collected and stored at −80°C. Protein concentration was determined using a BCA assay (Pierce). Samples were loaded onto 12% acrylamide gels (Bio-Rad) at 50 μg total protein per lane, and run at 120 V until the protein front was well focused at the beginning of the resolving gel, before band separation. The gels were transferred into fixative solution containing 10% acetic acid, 50% methanol (all from Fisher Scientific), and 40% water for 30 min, stained for a few seconds with Ponceau S (Boston Bioproducts, Inc.), then destained in fixative solution for 2–3 hours, until the protein band was clearly visible and the background minimal. The protein bands were excised, fragmented into 1-mm^3^-pieces, and stored in 1% acetic acid in water at 4°C overnight. The gel pieces were dried with 500 μl acetonitrile for 10 min, reduced with 10 mM DTT in 100 mM ammonium bicarbonate for 30 min at 56°C, dried with acetonitrile, alkylated with 55 mM iodoacetamide in 100 mM ammonium bicarbonate for 20 min at room temperature, and dried again with acetonitrile. The dried gel pieces were saturated with a solution of 13 ng/μl trypsin (Princeton Separations, Inc.) in 10 mM ammonium bicarbonate containing 10% acetonitrile, and incubated overnight in a thermo-mixer at 37°C and 400 rpm. Digests were extracted by adding a 1:2 solution of 5% formic acid in water and acetonitrile, followed by incubation at 37°C and 400 rpm for 15 min. Supernatant aliquots were collected and dried using a vacuum centrifuge.

eFASP was performed as previously described, with minor modifications [103]. Tissue samples were fragmented and incubated in a thermo-mixer for 10 min at 95°C and 600 rpm, in lysis buffer containing 4% SDS, 0.2% sodium deoxycholate, 50 mM Tris(2-carboxyethyl)phosphine hydrochloride, 100 mM ammonium bicarbonate, pH 8. The sample was further homogenized on ice using a sonicator, centrifuged at 14,000×g, sonicated and pelleted again, and the supernatant was collected and stored at −80°C. For sample processing, 25 μl of tissue lysate were mixed with 200 μl exchange buffer, containing 8 M urea, 0.2% sodium deoxycholate, 100 mM ammonium bicarbonate, pH 8, and dispensed onto a Vivaspin 500 filter unit, 30 kDa MWCO (GE Healthcare), and centrifuged at 15,000×g. After two further washes with exchange buffer, the sample was alkylated by incubating for 1 hour at 37°C and 300 rpm with 100 μl buffer containing 8 M urea and 50 mM iodoacetamide in 100 mM ammonium bicarbonate, pH 8. After centrifugation for 10 min at 15,000×g, and two further washes with 200 μl exchange buffer each, the sample was washed three times with digestion buffer containing 0.2% sodium deoxycholate in 50 mM ammonium bicarbonate, pH 8. Trypsin was added in 100 μl digestion buffer at a ratio of 1:50 with the total protein in the sample, and the unit was incubated overnight at 37°C and 300 rpm. After centrifugation for 10 min at 15,000×g, the unit was washed twice with 50 μl of 50 mM ammonium bicarbonate, and all filtrate was collected. To remove residual detergent, 1 ml of ethyl acetate was added to the sample, sonicated for 10 seconds and centrifuged for 10 min at 16,000×g, and the upper organic layer removed. This process was repeated three times, then the aqueous samples were placed in a thermomixer at 60°C for 5 min, to remove residual ethyl acetate. The samples were then dried in a vacuum centrifuge and resuspended in 50% methanol several times to remove residual salts.

### LC-MS/MS and data analysis for protein identification

Protein extract digests were analyzed on an impact HD Qq-Time-Of-Flight (Qq-TOF, Bruker Corporation, Billerica, MA) with a CaptiveSpray ion source (Bruker-Michrom, California, USA). The mass spectrometer was coupled to an Ultimate 3000 nano-LC system (Dionex, California, USA) fitted with an in-house packed C18 column (500 mm x 100 μm, 2.5 μm beads, XSelect, Waters Corp., Milford, MA). Solvent A was 0.1% formic acid in water, and solvent B was 0.1% formic acid in acetonitrile. The following gradient conditions were used: t = 0–5 min, 5% solvent B; t = 245 min, 40% solvent B; t = 248–262 min, 80% solvent B; t = 265 min, 5% solvent B. The flow rate was 1 μL/min. Tandem mass spectra were acquired at an intensity-dependent rate of 4-20Hz between precursor scans.

LC-MS/MS spectra were initially analyzed in DataAnalysis 4.2 (Bruker). Compounds were detected based on an AutoMSn intensity threshold of 10^4^ and exported to a Mascot generic file (MGF) using the Protein Analysis function. The MGFs from individual runs were imported into ProteinScape 3.1 (Bruker), combined, and proteins were identified from LC-MS/MS runs by searching the MS/MS spectra using a local Mascot (Matrix Science Inc, Boston, MA) server. All Mascot searches were performed using trypsin as the enzyme, up to two missed cleavages allowed, carbamidomethyl cysteine as a fixed modification, oxidized methionine and deamidated asparagine/glutamine as variable modifications, 10-ppm tolerance on the precursor and 0.5-amu tolerance on the product ions, and charge states of +1, +2, and +3. Identifications were evaluated using MudPIT scoring, with a minimum peptide score of 20. A decoy database search was performed to allow correction of protein identifications by a FDR threshold of 1%, 2%, and 5% probability, as described in Results and discussion. For Mascot searches based on public data, the NCBI database searches were limited to either *D. rerio* sequences or the Actinopterygii taxon. To construct databases built around our *A. leptorhynchus* assembly, “mostly likely” longest-ORF peptides candidates were generated from the assembly using TransDecoder (http://transdecoder.sourceforge.net/), both with and without ORF calling being biased towards ORFs with a recognizable domain in the Pfam protein families database [118] determined by HMMER 3.1 [119,120]. Redundant ORFs within sets were removed prior to compiling the ORF sets as Mascot databases.

## Availability of supporting data

This Transcriptome Shotgun Assembly project (BioProject ID PRJNA259518) has been deposited at DDBJ/EMBL/GenBank under the accession GBKR00000000. The version described in this paper is the first version, GBKR01000000. The mass spectrometry proteomics data have been deposited at the ProteomeXchange Consortium [121] via the PRIDE partner repository [122] with the dataset identifier PXD001285 and DOI 10.6019/PXD001285.

## Additional files

Additional file 1: Figure S1. Optimization of pre-assembly trimming and transcript reconstruction and the effect of FPKM filtering on the distribution of transcripts per contig and distribution of transcript length in the assembly. A. Relative to the total number of protein sequences in the *D. rerio* reference proteome set (41,112 sequences, black bar), similar coverage was achieved regardless of trimming strategy, although the “soft” trimming strategy provided a minor advantage over “hard” trimming, recovering 2.44% more transcripts with at least 80% sequence coverage (12,446 sequences, including antisense alignments, for soft trimming compared with 12,149 sequences for hard trimming). B. While the alternative transcript reconstruction methods reduced the overall number of transcripts, they produced similar number of overall contigs. C. The default (Butterfly) transcript reconstruction provided enhanced sensitivity, with 7.98% more sequences aligned than the next best reconstruction method (12,446 sequences covered with Butterfly compared with 11,526 with PasaFly). D. From the raw assembly, the number of transcripts per contig followed a power law, with many contigs having few transcripts and a few contigs having many (100–1000) transcripts. E. After filtering for transcripts with FPKM ≥ 1, the number of transcripts per contig was reduced to following a log distribution, where even though many contigs still had multiple transcripts, the number of transcripts was on the order of 10s. F. For the overall assembly, the size distribution of transcripts peaked between 1,000 and 10,000 nucleotides. G. When considering only the longest transcript per contig, the distribution was shifted to show most contigs’ longest transcript was closer to the assembler threshold of 200 nucleotides. H-I. After filtering for transcripts with FPKM ≥ 1, the distribution of transcripts in the assembly was distributed around 1,000 nucleotides (H), which was roughly maintained after considering only the longest transcript per contig (I). Additional file 2: Table S1. Assembly transcripts with best sense alignment per contig to *D. rerio* protein sequences (UniProt ID and accession), BLAST statistics, sequence coverage of target protein sequence, and description of *D. rerio* subject protein sequence. Additional file 3: Table S2. Assembly transcripts with best alignment per contig to Actinopterygii protein sequences (UniProt ID and accession), BLAST statistics, sequence coverage of target protein sequence, and description of *D. rerio* subject protein sequence. Shown are only BLAST hits that covered 80% or more of the target protein sequence. Additional file 4: Table S3. Significantly enriched GO terms based on FPKM ranking of “complete” transcripts (≥80% sequence coverage of homologous *D. rerio* protein), including genes enriched in each category, divided by cellular component, molecular function, and biological processes. ‘P-value’ is the enrichment p-value (unadjusted for multiple comparisons) computed according to the mHG or HG model. ‘FDR q-value’ is the multiple-testing corrected p-value using the Benjamini and Hochberg method, whereby, for the *i* ^th^ term (ranked according to p-value) the FDR q-value is (p-value *number of GO terms)/*i. N* is the total number of genes, *B* is the total number of genes associated with a specific GO term, *n* is the number of genes in the top of the user’s input list or in the target set when appropriate, and *b* is the number of genes in the intersection. Enrichment = (b/n) / (B/N). For each GO term, the list is shown of associated genes that appear in the optimal top of the list as determined by GOrilla (with percent of gene list considered from minimum hypergeometric scoring threshold shown in ‘Top%’ column). Each gene name is specified by gene symbol followed by a short description of the gene. Additional file 5: Table S4. Assembly transcripts with best alignment/gene to *D. rerio* ncRNA sequences, BLAST statistics, and sequence coverage of target ncRNA sequence. In general, alignments are sense, except where specified. Spreadsheet is divided into Kaushik et al. lncRNAs, antisense transcripts, lincRNA, mitochondrial rRNA, rRNA, SRP RNA, miRNA, snRNA, snoRNA, as well as, sense (intronic), sense (overlapping), and retained introns. Additional file 6: Figure S2. ORF prediction. The distribution of ORF lengths chosen by TransDecoder was similar overall, regardless of whether or not Pfam domains were used for guiding ORF selection (A without Pfam-biased ORF selection, B with Pfam-biased ORF selection). Similarly, overall FPKM filtering had little effect on ORF size distribution without Pfam-biased ORF selection (C) or including Pfam-biased ORF selection (D). E. Venn diagram of ORFs (no Pfam) vs FPKM ≥ 1 vs annotated protein coding transcripts (blastP). F. Venn diagram of ORFs (Pfam) vs. FPKM ≥ 1 vs annotated protein coding transcripts (blastP). G. Venn diagram of all unique BLAST hits from *D. rerio* reference set compared with unique BLAST hits from the set of ORFs found in transcripts with FPKM ≥ 1. H. Out of the 6,090 transcripts with FPKM ≥ 1 that contained ORFs of over 100 amino acids and did not align to a *D. rerio* protein in our reference sequence set, 3,505 had significant alignments when BLASTp-searched against the entire NCBI non-redundant (nr) protein sequence database. 3,273 of these alignments were to sequences from Teleostei species. Shown are the ten most enriched species and the number of ORFs (log-scale) that were matched to each. Additional file 7: Table S5.
*D. rerio* reference proteins with two or more distinct assembly contig ORFs that align to greater than 80% of the target *D. rerio* sequence. *D. rerio* reference proteins are shown by their UniProt accession and name followed by the corresponding assembly contigs. For *D. rerio* proteins with exactly two aligned contigs, the percent identity of the assembly contig ORFs relative to each other is shown. Also included are the ORF nucleotide sequences for all contigs in this table (shown is ORF of transcript with longest alignment for each contig). Additional file 8: Figure S3. Multiple ORFs align to uncharacterized *D. rerio* protein (A2BHK0). Little to no conservation was observed in the termini. Alignment performed with ClustalW2: *(asterisk) = fully conserved residue; : (colon) = conservation between groups of strongly similar properties (>0.5, Gonnet PAM 250 matrix); . (period) = conservation between groups of weakly similar properties (≤0.5). Additional file 9: Table S6. Protein IDs from database containing all ORFs chosen by TransDecoder (any size) with Mascot-assigned protein families, Mascot statistics, sequence coverage based on peptide assignments, BLAST results to *D. rerio* reference proteins (if found), sequence coverage of alignments, and protein sequence of identified ORFs.
