# Supplementary figures and images for: The central nervous system transcriptome of the weakly electric brown ghost knifefish (Apteronotus leptorhynchus): de novo assembly, annotation, and proteomics validation
Source: BMC Genomics. 2015 Mar 11;16(1):166. doi: 10.1186/s12864-015-1354-2 (PMC4424500; doi:10.1186/s12864-015-1354-2)

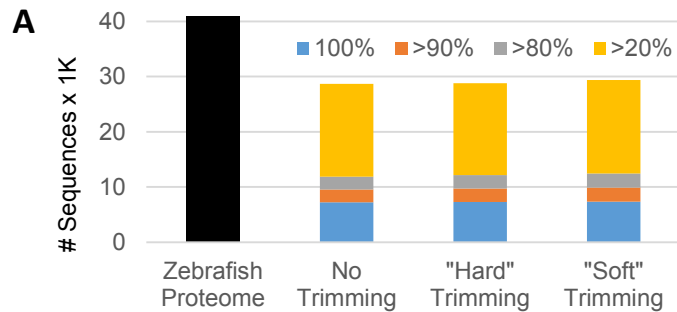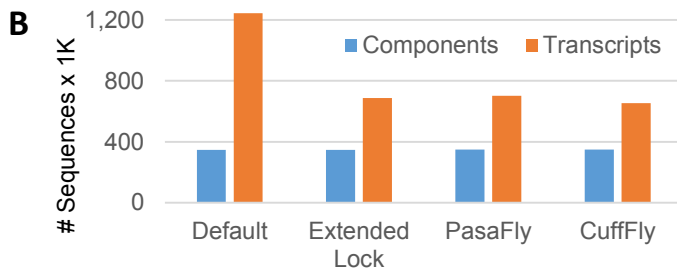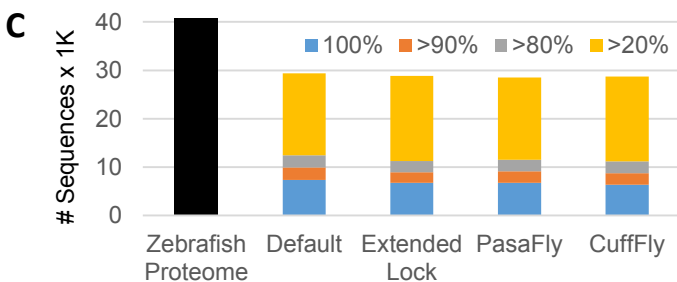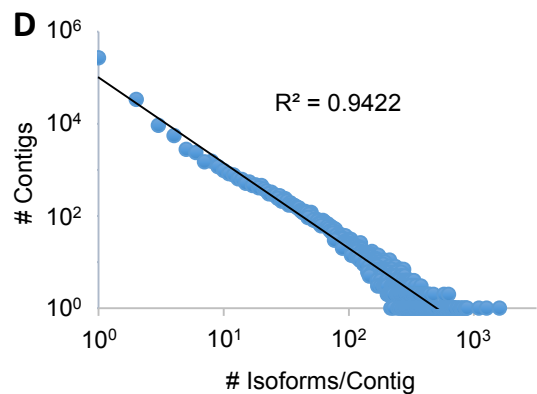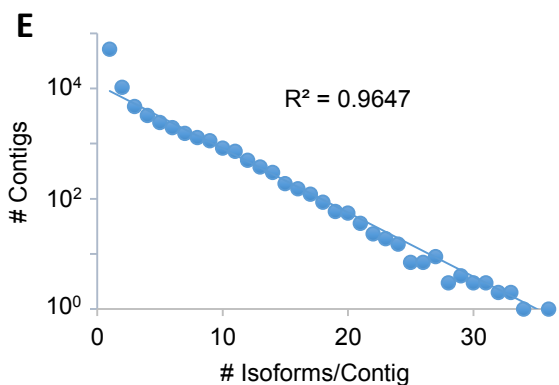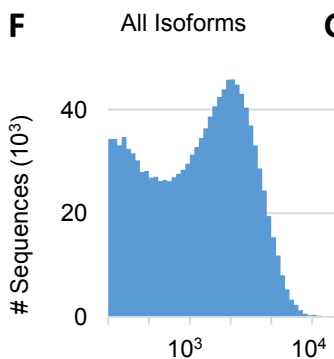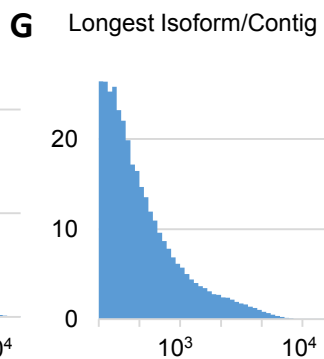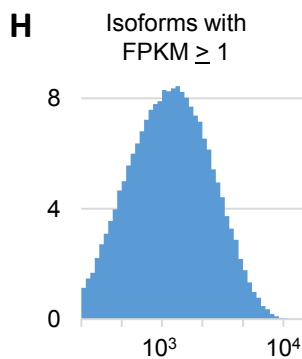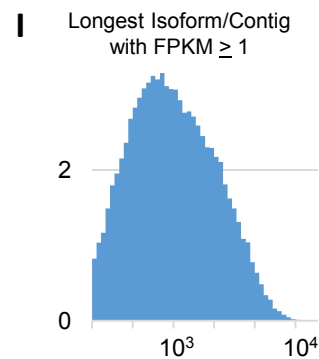

Supplement: Additional file 1: Figure S1. — Optimization of pre-assembly trimming and transcript reconstruction and the effect of FPKM filtering on the distribution of transcripts per contig and distribution of transcript length in the assembly. A. Relative to the total number of protein sequences in the D. rerio reference proteome set (41,112 sequences, black bar), similar coverage was achieved regardless of trimming strategy, although the “soft” trimming strategy provided a minor advantage over “hard” trimming, recovering 2.44% more transcripts with at least 80% sequence coverage (12,446 sequences, including antisense alignments, for soft trimming compared with 12,149 sequences for hard trimming). B. While the alternative transcript reconstruction methods reduced the overall number of transcripts, they produced similar number of overall contigs. C. The default (Butterfly) transcript reconstruction provided enhanced sensitivity, with 7.98% more sequences aligned than the next best reconstruction method (12,446 sequences covered with Butterfly compared with 11,526 with PasaFly). D. From the raw assembly, the number of transcripts per contig followed a power law, with many contigs having few transcripts and a few contigs having many (100–1000) transcripts. E. After filtering for transcripts with FPKM ≥ 1, the number of transcripts per contig was reduced to following a log distribution, where even though many contigs still had multiple transcripts, the number of transcripts was on the order of 10s. F. For the overall assembly, the size distribution of transcripts peaked between 1,000 and 10,000 nucleotides. G. When considering only the longest transcript per contig, the distribution was shifted to show most contigs’ longest transcript was closer to the assembler threshold of 200 nucleotides. H-I. After filtering for transcripts with FPKM ≥ 1, the distribution of transcripts in the assembly was distributed around 1,000 nucleotides (H), which was roughly maintained after considering only the longest transcript pe [file 12864_2015_1354_MOESM1_ESM.pdf]

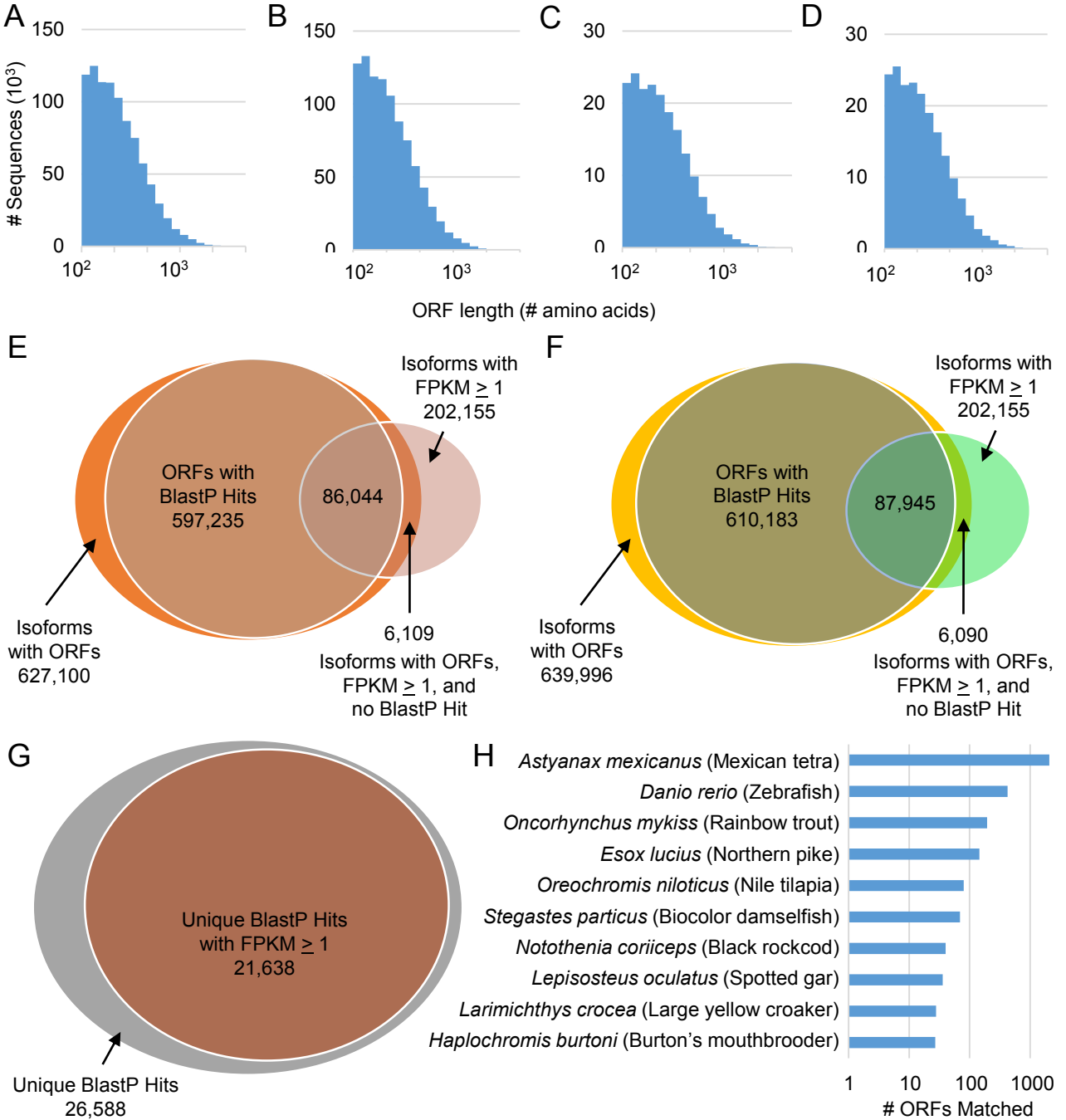

Supplement: Additional file 6: Figure S2. — ORF prediction. The distribution of ORF lengths chosen by TransDecoder was similar overall, regardless of whether or not Pfam domains were used for guiding ORF selection (A without Pfam-biased ORF selection, B with Pfam-biased ORF selection). Similarly, overall FPKM filtering had little effect on ORF size distribution without Pfam-biased ORF selection (C) or including Pfam-biased ORF selection (D). E. Venn diagram of ORFs (no Pfam) vs FPKM ≥ 1 vs annotated protein coding transcripts (blastP). F. Venn diagram of ORFs (Pfam) vs. FPKM ≥ 1 vs annotated protein coding transcripts (blastP). G. Venn diagram of all unique BLAST hits from D. rerio reference set compared with unique BLAST hits from the set of ORFs found in transcripts with FPKM ≥ 1. H. Out of the 6,090 transcripts with FPKM ≥ 1 that contained ORFs of over 100 amino acids and did not align to a D. rerio protein in our reference sequence set, 3,505 had significant alignments when BLASTp-searched against the entire NCBI non-redundant (nr) protein sequence database. 3,273 of these alignments were to sequences from Teleostei species. Shown are the ten most enriched species and the number of ORFs (log-scale) that were matched to each. [file 12864_2015_1354_MOESM6_ESM.pdf]
