# Supplementary material for: The central nervous system transcriptome of the weakly electric brown ghost knifefish (Apteronotus leptorhynchus): de novo assembly, annotation, and proteomics validation
Source: BMC Genomics. 2015 Mar 11;16(1):166. doi: 10.1186/s12864-015-1354-2 (PMC4424500; doi:10.1186/s12864-015-1354-2)
Supplement: Additional file 8: Figure S3. — Multiple ORFs align to uncharacterized D. rerio protein (A2BHK0). Little to no conservation was observed in the termini. Alignment performed with ClustalW2: *(asterisk) = fully conserved residue; : (colon) = conservation between groups of strongly similar properties (>0.5, Gonnet PAM 250 matrix); . (period) = conservation between groups of weakly similar properties (≤0.5). [file 12864_2015_1354_MOESM8_ESM.pdf]

```
A2BHK0_DANRE            ------------------------------------------------------------
comp167859_c1_seq2      MAGKVNKEDTSSLHLGNTPVNNGDKTSKRKISDVDTQAQHHPDKSAVPVMQKHISDPHCT 60
comp168050_c0_seq1      ------------------------------------------------------------
comp177116_c0_seq5      ------------------------------------------------------------
comp165668_c0_seq4      ------------------------------------------------------------

A2BHK0_DANRE            -----------------------------------------------MYQLSQPEMITGK 13
comp167859_c1_seq2      VPESSTCVTMETTEDCLVHKHQIMKDENLSNDGEEQVKIDLLDAINDDQTLGNNARKSLR 120
comp168050_c0_seq1      ------------------------------------------------------MFQALI 6
comp177116_c0_seq5      -------------------------------------------------------CSSDL 5
comp165668_c0_seq4      ----------------------------------------MFLTSKEIMNSEYKESDQDL 20

A2BHK0_DANRE            YYYVLETGRTGKLRMEILKHLLKQRSHLEEVYSVNQCDVILVLCP---ITSRAGTDIDAA 70
comp167859_c1_seq2      KYFTFVPGNTFNSHETFKTRLQQEVPHLQEVFTEEECDFFVLFCP---VVSRAGTDIEAA 177
comp168050_c0_seq1      RVFVMVFGNTMKSHESFVKNLKQSF-NLEEVSSANSCDVILAFVS---VVSRVGTDIDAA 62
comp177116_c0_seq5      EFKYFVAETNKKPPDSVRKAVHKRIPQLLEVSTVEECDIILVFYLTEPISSWSETDIAEA 65
comp165668_c0_seq4      KGFFTIQDPENAINLNFLEFFHDVRPDLKEVPSVEKSDLVLVFCY----VSEIQRDIEKA 76
                                        .   . .   .* ** : :..*..: :       *    **  *

A2BHK0_DANRE            LNKLN--KLSASKPAIFMVLHHTSDPYKVVPDSGRFIQ-GMNMLTVDILFYEDVGLLNCI 127
comp167859_c1_seq2      LMKLNSFKTSGSKPAVIVVLHHTFDPQRIVPDSRRSVN-RMDTITVDCLFYEDTGLLQCP 236
comp168050_c0_seq1      LQKIP-----DGRPVLLVVLHHTFDPYHIAPQSRLCVMNRSDVFAVDCLYYENHGLLKSL 117
comp177116_c0_seq5      MQTIS--CITGTKPVVLMVFHHTVNPEYTPPDSRRWVT-MSDTFSVDCLFAEDKGLLPCP 122
comp165668_c0_seq4      LQKLN--SVAASKPAVLVVLHYTSDPKNIS-DSTKYVN-RWDTITVDCLISEDKTLLKCS 132
                        : .:        :*.:::*:*:* :*      :*   :    : ::** *  *:  ** .

A2BHK0_DANRE            MNDEALGRVVRCFKPQ-------------------------------------------- 143
comp167859_c1_seq2      KNDEAIEQIKEWIKSQVHEEQETGYL---------------------------------- 262
comp168050_c0_seq1      HNDNALKAVKQHLRENVLS----------------------------------------- 136
comp177116_c0_seq5      KNDEAFYEFFKWLESKVFVS---------------------------------------- 142
comp165668_c0_seq4      KNDELLSRFAKQITSEVFSEVKEEVTPLLIWHHTTAIICVCFLLLKETYSFYMIYFLWPI 192
                         **: :  . . :  :

A2BHK0_DANRE            ------------------------------------------------------------
comp167859_c1_seq2      -------------FYPYKIIEYGWATFKFLFSFVTRSNTYSENPASESMKQMSETVVATK 309
comp168050_c0_seq1      ---------------------------KKHLTFNTKE-SESVCPMTPTYKLIYIVIIFTF 168
comp177116_c0_seq5      --------------------------IRQRFA---------------------------- 148
comp165668_c0_seq4      FCSFKDQAHCTAKQLPSPFLHKYFADFIGQVPQCYVEILSGLHEQRPDLQEVATVEKCDV 252

A2BHK0_DANRE            -------------------------------
comp167859_c1_seq2      LRLVLLGRTGSGKSA---------------- 324
comp168050_c0_seq1      IYIFYSLKNIL-------------------- 179
comp177116_c0_seq5      -------------------------------
comp165668_c0_seq4      ILAFFISRPGSGIEAALKQIDSHTGSKPVVL 283
```
